# Strengthening access to refractive error services in a remote district of Assam, India

**Published:** 2024-05-15

**Authors:** Shubhrakanti Bhattacharya, Franklin Daniel, Elizabeth Kurian

**Affiliations:** 1 Senior Manager, Programme Development: Mission for Vision, Kolkata, India.; 2 Lead, Vision Centre Programme: Mission for Vision, Hyderabad, India.; 3 Chief Executive Officer: Mission for Vision, Mumbai, India.

**The vision centre approach has increased access to refractive error services at the level of primary eye care in a remote area in India.**

**Figure:**
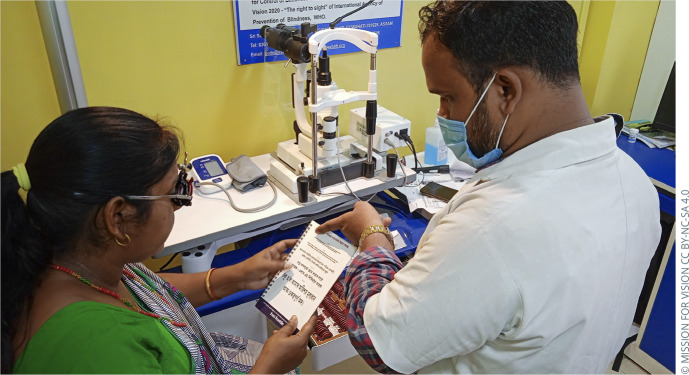
Near vision examination of a patient at Barpeta Vision Centre, Assam. india

Refractive error is one of the major causes of visual impairment amongst all age groups and genders in India.^1^ Key factors include insufficient awareness, a shortage of skilled human resources, and a lack of adequate evidence about effective approaches to address refractive error, especially in remote settings.

The Barpeta Vision Centre was set up in February 2022 as a partnership between Mission Jyoti, a Mission for Vision initiative, and Sri Sankaradeva Nethralaya, an eye hospital in Guwahati in the north-eastern state of Assam, India. Its aim is to address the burden of visual impairment in Barpeta, a remote district of Assam, by offering affordable primary eye care services.

Before the vision centre was established, people with refractive errors could access services only during the cataract screening camps organised by Sri Sankaradeva Nethralaya, or by traveling 90 km to the nearest tertiary eye centre in Guwahati. People who needed spectacles had to buy them from local optical outlets at rates that were unaffordable for the majority.

The vision centre serves a population of about 50,000 and is located in rented premises with an area of 340 square feet, in the market area of Barpeta town. People from surrounding villages, within a radius of 15 to 20 km, come to Barpeta market for various needs and can then also access eye care facilities at the vision centre. The vision centre also organises two or three community camps each month in villages within a radius of 5 to 8 km from Barpeta. During these camps, screening, identification, and referral services are provided. Patients identified with cataract are referred to the tertiary eye centre in Guwahati for surgery, and those who require spectacles and/or further examination are referred to the vision centre in Barpeta.

For the local communities, the distance to be covered and travel time required to access services such as basic eye examination, refraction test, identification of cataract and other conditions, and dispensing of spectacles have therefore been considerably reduced.

## Human resources

The vision centre is staffed by an optometrist and two female community health workers. It is overseen by a co-ordinator who is responsible for managing other community activities conducted by Sri Sankaradeva Nethralaya in the area.

The community health workers and the co-ordinator are trained by, and affiliated to, the tertiary eye facility in Guwahati. The clinical staff are trained as part of a university-recognised course at Sri Sankaradeva Nethralaya.

The community health workers conduct door-to-door screening and refer patients as needed; interact with various stakeholders such as heads of schools, teachers, staff of primary health centres, and local community leaders; and organise community eye camps and vision screening programmes in schools. The female health workers interact with women during the door-to-door visits, talk to them about the vision centre and its services, and create awareness among women about the need for eye care for themselves and their children.

## Services

The services offered by the vision centre include the following:

- door-to-door screening within the catchment area, with referrals as needed
- counselling of patients
- refraction error services and dispensing of spectacles
- diagnosis of cataract and other common conditions, and referral to the hospital in Guwahati
- postoperative follow-ups
- school eye health services, which include screening school children in the catchment area, providing free spectacles to children identified with uncorrected refractive error, and referring those who require further diagnosis and treatment to the base hospital
- eye health information and education services to raise community awareness of the need for regular eye check-ups.

The centre is compliant with the World Health Organization's guidelines for conducting vision and eye screenings in community and primary care settings ^2^ and is equipped to assess refractive error and cataract.

## Provision of spectacles

The vision centre stocks spectacles varying in quality and price. The lowest-priced spectacles cost INR 450 (approximately US $5) compared to INR 750 (approximately US $9) at the local optical outlets; this makes them more affordable to people on lower incomes. The most expensive pair at the vision centre would cost INR 900 (approximately US $11) compared to INR 3,000 (approximately US $38) in shops outside.

Spectacle lenses are made and fitted at the grinding and fitting workshop at the base hospital and delivered to the patients through the vision centre, with a turnaround time of three to four days.

## Financial viability

The vision centre charges each new patient a registration fee of INR 100 towards eye examination, diagnosis, and referral (if required). This is valid for one month, during which period additional visits are not charged. The registration fee is waived for patients who cannot afford to pay it.

The financial viability of the vision centre is ensured through the registration fee and the sale of spectacles. These income streams meet the direct running cost of the vision centre and contribute to the sustainability of services. Apart from the registration fee, there are no other charges for patients for eye care services at the vision centre. The costs are covered by Sri Sankaradeva Nethralaya, with funds generated from patients opting to pay for surgery packages being used to cross-subsidise patients who cannot afford to pay. Thus, the cost of the services is never a burden on those who cannot afford them.

## Trends in access to services at the vision centre

Over the 23 months since it was established, the vision centre has shown a steady increase in uptake of services. Starting with 42 people in February 2022, the vision centre now serves around 460 people each month. Notably, 85% of those prescribed spectacles prefer to buy them from the vision centre, with the rest choosing to procure them from other optical outlets.

Increasing women's access to eye care is one of the key aims of the vision centre. Of the beneficiaries who were screened at the vision centre in Barpeta and its community camps, 58% were women. About 56% of those who received spectacles and 57% of those who underwent surgery through the vision centre were women.

## Networks

The vision centre has established networks with local government functionaries, officials from the health, education, and social welfare departments, and key stakeholders at various levels to ensure increased uptake of services. Whereas the vision centre personnel have close links with organisations at the local panchayat level, the base hospital team is supported by networking with the district-level government authorities. As we have seen, the centre's female health workers closely interact with various stakeholders in the community.

## Success factors

Even though the Barpeta Vision Centre has been operational for less than two years, an increasing number of people, particularly women, have access to quality primary eye care. This has been achieved by

- offering comprehensive eye care services
- strengthening the eye health workforce in a remote location through the provision of trained personnel
- engaging with people and communities
- prioritising the provision of services to vulnerable populations, primarily children and women
- integrating eye health into the wider health system through networking and referrals
- fostering linkages with the tertiary facility, local community-based organisations, and NGOs.

Over the past 23 months, this initiative has highlighted the value and utility of the vision centre approach in providing people-centred, affordable, and comprehensive eye care, including refractive error care, in a remote area. Other instances of successful outcomes of the vision centre approach in India have also been documented. ^3^

## References

[1] Government of India, Ministry of Health and Family Welfare. National blindness and visual impairment survey 2015–2019 [Internet]. Available from: https://tinyurl.com/yzz5zfmm

[2] World Health Organization. Department of Noncommunicable Diseases. Vision and eye screening implementation handbook [Internet]. Geneva: World Health Organization; January 2024. Available from: https://tinyurl.com/36mxbv4p

[3] Khanna RC Sabherwal S Sil A Gowth M Dole K Kuyyadiyil S. Primary eye care in India– the vision centre model. Indian J Ophthalmol. 2020;68(2):333-9. 31957722 10.4103/ijo.IJO_118_19 PMC7003605

